# Impact of Grassland Management System Intensity on Composition of Functional Groups and Soil Chemical Properties in Semi-Natural Grasslands

**DOI:** 10.3390/plants14152274

**Published:** 2025-07-24

**Authors:** Urška Lisec, Maja Prevolnik Povše, Miran Podvršnik, Branko Kramberger

**Affiliations:** 1 Department of Grassland and Fodder Production, Faculty of Agriculture and Life Sciences, University of Maribor, Pivola 10, 2311 Hoče, Slovenia; miran.podvrsnik@um.si (M.P.); branko.kramberger@um.si (B.K.); 2 Department of Animal Science, Faculty of Agriculture and Life Sciences, University of Maribor, Pivola 10, 2311 Hoče, Slovenia; maja.prevolnik@um.si

**Keywords:** grassland biodiversity, management intensity, composition of functional groups, soil chemical and physical properties, carbon and nitrogen storage, soil C:N ratio, sustainability

## Abstract

Semi-natural grasslands are some of the most species-rich habitats in Europe and provide important ecosystem services such as biodiversity conservation, carbon sequestration and soil fertility maintenance. This study investigates how different intensities of grassland management affect the composition of functional groups and soil chemical properties. Five grassland management systems were analyzed: Cut3—three cuts per year; LGI—low grazing intensity; CG—combined cutting and grazing; Cut4—four cuts per year; and HGI—high grazing intensity. The functional groups assessed were grasses, legumes and forbs, while soil samples from three depths (0–10, 10–20 and 20–30 cm) were analyzed for their chemical properties (soil organic carbon—SOC; soil total nitrogen—STN; inorganic soil carbon—SIC; soil organic matter—SOM; potassium oxide—K_2_O; phosphorus pentoxide—P_2_O_5_; C/N ratio; and pH) and physical properties (volumetric soil water content—VWC; bulk density—BD; and porosity—POR). The results showed that less intensive systems had a higher proportion of legumes, while species diversity, as measured via the Shannon index, was the highest in the Cut4 system. The CG system tended to have the highest SOC and STN at a 0–10 cm depth, with a similar trend observed for SOC_stock_ at a 0–30 cm depth. The Cut4, HGI and CG systems also had an increased STN_stock_. Both grazing systems had the highest P_2_O_5_ content. A tendency towards a higher BD was observed in the top 10 cm of soil in the more intensive systems. Choosing a management strategy that is tailored to local climate and site conditions is crucial for maintaining grassland stability, enhancing carbon sequestration and promoting long-term sustainability in the context of climate change.

## 1. Introduction

Agroecosystem management improves soil carbon sequestration by converting CO_2_ into stable organic carbon (SOC) and contributes to climate regulation by improving SOC density, distribution and the stability of microaggregates [1]. Grasslands contribute to climate change mitigation by storing atmospheric CO_2_ as stable soil carbon, which is critical for soil health and agricultural productivity and improves the ecological resilience of ecosystems [1,2,3].

Grasslands store about one-third of the world’s terrestrial carbon, with the potential for soil carbon sequestration estimated at 2.3 to 7.3 billion tons of CO_2_ equivalent (CO_2_e) per year [4,5,6]. Globally, grassland restoration can sequester 6.8 billion tons of CO_2_e per year, 148–699 megatons of CO_2_e per year through improved grazing and a further 147 megatons through the sowing of legumes on pastures [4]. As 80% of European grasslands are below the saturation limit, there is considerable potential for sequestering SOC [7]. Organic carbon stocks in soil can be increased or decreased with more intensive management due to complex interactions between grazing, carbon input into the soil and decomposition processes [8]. However, the sequestration of SOC and nitrogen through improved management and restoration is influenced by various environmental factors—including land management, climate change [9], animal type, fertilization regime and botanical composition [2,10]—resulting in carbon sequestration rates between 0.11 and 3.04 Mg C ha^−1^ per year [11]. The aim of this study is to investigate how five different grassland management systems affect the composition of functional groups and the chemical and physical properties of soil to identify practices that support both the conservation of biodiversity and the sustainable functioning of ecosystems.

As a key factor in SOC dynamics, moderate grassland management [12] influences both nutrient status and species composition in above- and belowground functions [13]. Pastures and meadows, which cover 68% of the world’s agricultural land, have a significant impact on the variability in soil nutrients [14], especially on SOC, soil total nitrogen (STN) and phosphorus content, due to the different biomass yields in grassland systems [2]. However, the effects of grazing intensity on SOC are climate-dependent (e.g., temperature and precipitation), so the same level of grazing intensity can have different effects on SOC levels in different climatic zones [10].

Controlled and moderate grazing with high grazing densities significantly increases the SOC of topsoil [15], on average by 21% [3,16], and improves the STN stock compared to grazing with low densities and cutting [3,14]. Controlled grazing intensity also promotes better plant growth [16] and increases root carbon content in the driest and wettest sites, while it decreases root carbon content in areas with medium rainfall (400–850 mm) [17], with rainfall having a particular effect on grass productivity and net primary production [18]. Frequent grazing leads to a higher deposition level of herbivore excrement compared to low frequent grazing and cutting, with SOC concentrations increasing by 12% with higher cutting frequency [19]. Cutting can lead to higher net carbon uptake through photosynthesis than grazing, depending on the biomass harvested and returned to the soil [16].

In the past, the diversity of semi-natural grasslands has been negatively affected by changes in land management and utilization [20]. The management system significantly influences plant diversity and is crucial for explaining the carbon content in soil in experiments. More extensive management provides a greater opportunity for seedling regeneration and has the potential to increase the abundance of less desirable species [21,22]. Combined management with cutting and late grazing can maintain species diversity for dairy cattle nutrition [23,24] and improve seedling regeneration [22,25], whereas grazing alone reduces plant richness [23]. The authors have shown that the combined effects of cutting and grazing increase rhizodeposition and the microbial availability of carbon substrates, while soil organic matter (SOM) mineralization rates decrease, thus indicating improved microbial energy efficiency [26].

High plant diversity increases soil carbon stocks and facilitates deep sequestration [27] while also improving SOC storage by increasing root biomass, exudates and microbial growth, thus ensuring high biodiversity and carbon sequestration [4,28]. Moderately and intensively managed grasslands have a faster root turnover than less intensively managed grasslands [29]. The efficiency of SOC stabilization through root carbon supply is about five times greater than that through aboveground supply [30].

Functional groups influence the stability, productivity and nutrient dynamics of ecosystems [31]. Grasslands dominated by C3 and C3-C4 species are more sensitive to the loss of SOC when grazing intensity is higher [32]. Soil carbon sequestration is likely to be associated with greater grass cover, which increases carbon input through roots that efficiently fix nitrogen in the soil [3]. Compared to other plant species, legumes have a higher CO_2_ production level per unit biomass, contribute to biological nitrogen fixation and help to reduce greenhouse gas emissions [33] and dependence on mineral fertilizers [34]. When grasses and other forage crops are equally represented, legumes can increase SOC by 7–17%. At medium to high grazing intensity, the dominance of grasses increases SOC [35]. Grasses effectively take up nitrogen from legumes via their fibrous roots due to their larger root surface [36]. Driven by legume nitrogen fixation and transfer through litter, dead roots and exudates, ecosystem demand for nitrogen in soil can promote grass root growth and access to legumes and non-symbiotic nitrogen sources [33]. The increased input through the roots and distribution to deeper soil layers increases the particulate organic matter content in the subsoil [4].

Traditional grassland management systems need to be adapted to changing climatic conditions and pressures on agricultural production. An important aspect of this effort involves finding a balance between productivity and sustainability. Research has shown that combined grazing and cutting systems offer opportunities to improve carbon and nitrogen storage while enabling more efficient nutrient utilization [37], higher root biomass and a greater conservation of botanical diversity [23,38]. This article aims to analyze the effects of five different grassland management intensity systems on the botanical communities of semi-natural grasslands and their effects on the stocks of soil chemical (SOC; STN; SOM; STC—soil total carbon; inorganic soil carbon—SIC; potassium oxide —K_2_O; phosphorus pentoxide—P_2_O_5_; C/N ratio; and pH) and physical (volumetric soil water content—VWC; bulk density—BD; and porosity—POR) properties at different soil depths. Furthermore, we hypothesize that the proportion of legumes will decrease with increasing management intensity. Among the systems analyzed, we expect the combined cutting and grazing (CG) system to be the most effective, as it is expected to promote higher SOC and STN stock.

Furthermore, this study aims to improve the understanding of how different grassland management strategies affect soil carbon and nitrogen sequestration while supporting sustainable grassland use. We also assess how these systems can be adapted to reduce negative environmental impacts, such as greenhouse gas emissions, nutrient leaching and soil erosion, through improved nutrient and livestock management and the maintenance of vegetation cover. Given the limited knowledge of how the intensity and combination of grassland systems affect plant functional groups and soil ecosystem functions, this research fills a critical knowledge gap for Slovenia and the entire European region and provides a basis for more sustainable grassland management practices.

## 2. Results

### 2.1. Functional Group Composition and Diversity Indices in Different Management Systems

According to the results, the intensity of the management system had different effects on the number and proportion of plants (Table 1). The less intensive management systems had a significantly higher proportion of legumes (LGI = 15.0% and Cut3 = 13.5%), compared to the more intensive farming systems (Cut4 = 6.6% and HGI = 5.4%) (*p* < 0.001), with the CG system having an intermediate position (CG = 10.4%). A significantly higher proportion of grasses was found for the CG (63.0%), Cut4 (62.9%) and HGI systems (61.0%) compared to less intensive systems (Cut3 = 56.0% and LGI = 45.5%). Although the proportion of forbs in the different management systems varied between 26% and 37%, the management system had no significant effect on their overall proportion (Table 1). The Shannon diversity index, which represents diversity considering evenness, was significantly higher for Cut4 than for the CG, LGI and Cut3 management systems, with the lowest value found for HGI. Although the highest species richness was observed in the LGI and Cut3 systems, the differences were not statistically significant (Table 1).

### 2.2. The Relationship Between Different Functional Groups and the Storage of Different Chemicals Properties in the Soil

**Figure 1:**
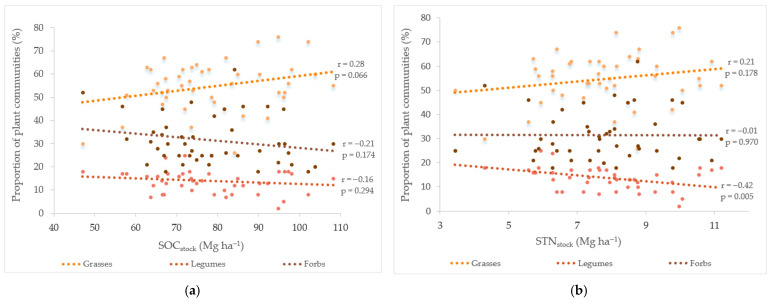

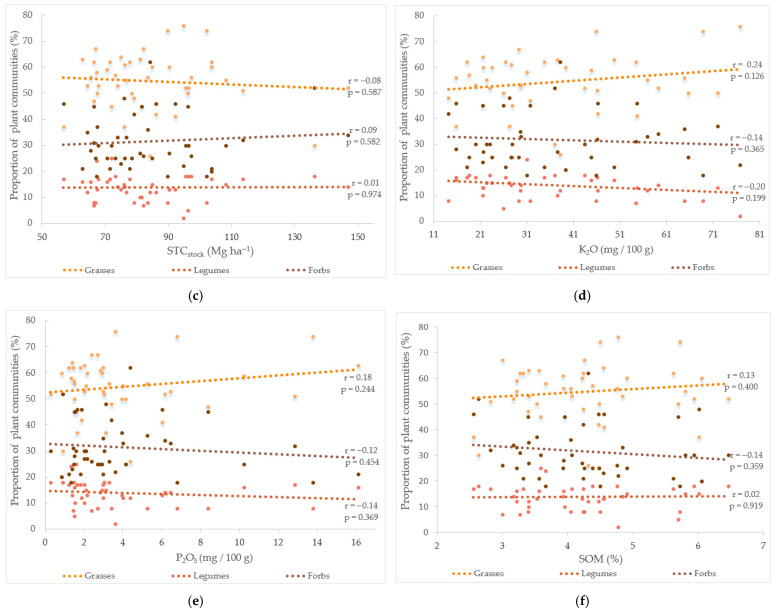
The correlations (r) of some of the soil chemical properties at a 0–30 cm depth with the proportion of functional groups (grasses, legumes and forbs). The parameters SOC_stock_ (**a**), STN_stock_ (**b**), STC_stock_ (**c**), K_2_O (**d**), P_2_O_5_ (**e**) and SOM (**f**) denote the total amounts of soil organic carbon (SOC), total nitrogen (STN), total carbon (STC), potassium oxide (K_2_O) and phosphorus pentoxide (P_2_O_5_), respectively, within the 0–30 cm soil layer and with respect to the mean organic matter (SOM).

Figure 1 shows the Pearson correlations between the different plant communities and the storage of different soil chemicals properties. A higher proportion of legumes was associated with a lower value of STN_stock_ (r = −0.42; *p* = 0.005), and the proportion of grasses tended to be positively associated with SOC_stock_ (r = 0.28; *p* = 0.066). No statistically significant correlations were found between the different proportions of the plant groups and SOM or STC_stock_, P_2_O_5_ or K_2_0, which indicates that the ratios between the functional groups are only minimally influenced by the soil properties.

### 2.3. The Effects of the Different Intensities of Management Systems on the Physical and Chemical Properties of Soil

The effects of the different management systems and soil depths on the physical and chemical properties of soil are shown in Table 2. Soil management systems had no significant effect on soil physical properties, while soil depth affected VWC, porosity and BD. No significant interaction effects between the management system and soil depth were observed for any of the physical properties. Further analysis (Table 3) shows the effects of management systems within each soil depth and vice versa. The tendency towards a higher BD was observed in the first soil depth for the CG system, Cut4 and HGI, followed by LGI and Cut3. Neither porosity nor VWC were affected by the intensity of grassland management at the different soil depths.

**Figure 2:**
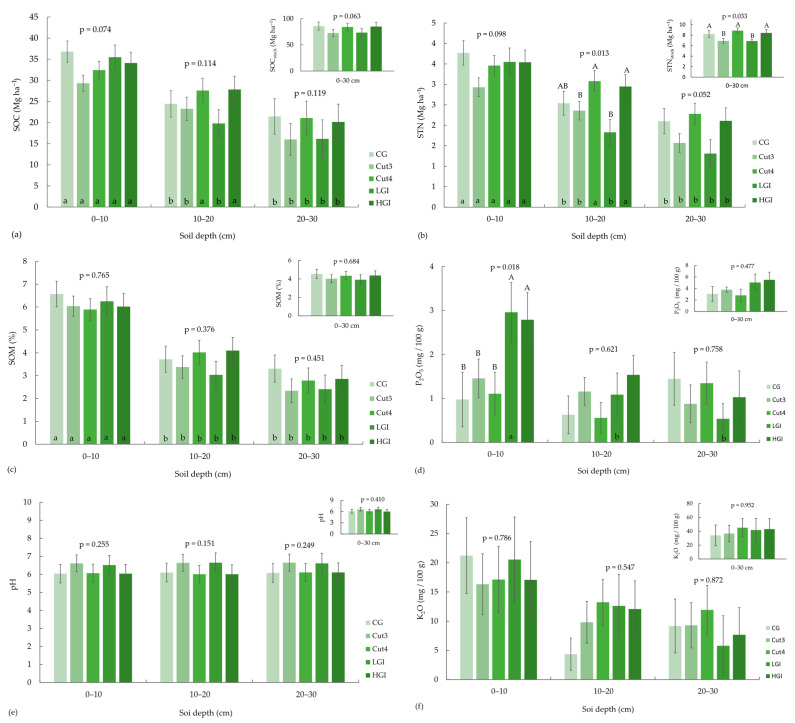

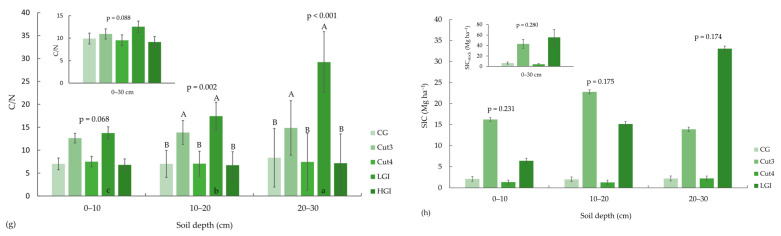
Soil chemical properties under five different grassland management systems at three depths (0–10, 10–20 and 20–30 cm) and stock at 0–30 cm depth (presented as means ± standard errors). Horizontal distribution of SOC (**a**), STN (**b**), SOM (**c**), P_2_O_5_ (**d**), K_2_O (**f**), SIC (**h**) concentrations, C/N (**g**) and pH (**e**) at different depths and 0–30 cm under five different grassland management systems: CG—combined cutting and grazing; Cut3—three cuts per year; Cut4—four cuts per year; LGI—low grazing intensity; HGI—high grazing intensity. (A, B): Different letters indicate significantly different means between management systems within each depth based on the Bonferroni test with *p* < 0.05. (a, b, c): Different letters denote significantly different means between depths within each management system based on the Bonferroni test with *p* < 0.05. Soil chemical properties: soil organic carbon—SOC; soil total nitrogen—STN; soil inorganic carbon—SIC; soil organic matter—SOM; potassium oxide—K_2_O; phosphorus pentoxide—P_2_O_5_; C/N—carbon/nitrogen ratio.

The management system influenced four (SIC, STN, pH, C/N) of the nine soil chemical properties. The effects of the management system × soil depth interaction significantly influenced only C/N. In our study, the effects of the management system on the chemical properties were further determined separately at each depth of the soil and vice versa (Figure 2). The trend of the highest SOC (*p* = 0.074) was observed at the first soil depth managed with the CG system (36.80 Mg ha^−1^), followed by LGI (35.48 Mg ha^−1^), HGI (34.10 Mg ha^−1^), Cut4 (32.43 Mg ha^−1^) and Cut3 (29.31 Mg ha^−1^). The same trend (*p* = 0.098) of the highest STN concentration was observed in the CG system at the first soil depth, followed by the management systems in the same order as that seen for SOC. At the second soil depth, STN concentration was significantly higher (*p* = 0.013) in Cut4 (3.08 Mg ha^−1^) and HGI (2.95 Mg ha^−1^), followed by CG (2.54 Mg ha^−1^), than in Cut3 (2.36 Mg ha^−1^) and LGI (1.83 Mg ha^−1^). A similar trend was observed for STN at the third depth, with the lowest values in the Cut3 (1.57 Mg ha^−1^) and LGI systems (1.31 Mg ha^−1^). At the first soil depth, the values for P_2_O_5_ were significantly higher (*p* = 0.018) for both grazing systems, the LGI (2.96 mg/100 g) and HGI systems (2.79 mg/100 g), compared to the Cut3 (1.46 mg/100 g), Cut4 (1.11 mg/100 g) and CG systems (0.98 mg/100 g). The concentrations of SIC were higher in the less intensive systems Cut3 and LGI at all soil depths, but there was no significant difference between them. The management system had no effect on pH or K_2_O. Although the highest pH was observed in the LGI and Cut3 systems, the differences were not statistically significant. The C/N ratio differed at the second depth (*p* = 0.002), showing a significantly higher C/N ratio in the LGI and Cut3 systems (17.43 and 13.86, respectively) and at the third soil depth (*p* < 0.001) in the LGI and Cut3 systems (29.22 and 14.86, respectively).

Compared to LGI (6.97 Mg ha^−1^) and Cut3 (6.99 Mg ha^−1^), the management systems with higher intensity, such as Cut4 (8.90 Mg ha^−1^), HGI (8.46 Mg ha^−1^) and CG (8.27 Mg ha^−1^), had significantly higher STN_stock_ (Figure 2, *p* = 0.033). The trend of the highest concentration of SOC_stock_ (*p* = 0.063) was observed in the CG (85.86 Mg ha^−1^) system, followed by HGI (85.03 Mg ha^−1^), Cut4 (84.19 Mg ha^−1^), LGI (73.43 Mg ha^−1^) and Cut3 (72.56 Mg ha^−1^). The concentrations of SOM for the individual management systems follow the same order as those for SOC, but they were not statistically significant.

Soil depth influenced seven of the nine chemical properties (SOC, STC, STN, P, K, SOM and C/N) (Table 2). SOC, STN, SOM and POR were affected by depth in all five grassland management systems, with higher concentrations in the topsoil compared to the subsoil (Figure 2, Table 3). SOC and STN showed a highly significant effect of depth, with concentrations significantly higher at the first two depths than at the third depth. Soil depth also had a significant effect on BD in all five systems, which increased with depth. The C/N ratio was significantly influenced by depth only in the LGI system, with the highest value in the deepest layer (29.22) compared to the second (17.43) and first (13.74) layers. In contrast, soil depth had no effect on pH, K_2_O or SIC (Figure 2). Soil pH showed higher values in the less intensive systems LGI and Cut3 than in the more intensive systems, and this was the same at all individual depths from 0 to 30 cm (Table 2).

## 3. Discussion

### 3.1. Functional Groups in Relation to Intensity of Grassland Management

The balance between grassland biodiversity and agricultural productivity often involves trade-offs, especially under site-specific environmental and management conditions. This study investigated how five grassland management systems influence the composition of functional groups and soil chemical properties at different depths. These findings are relevant for sustainable agri-environmental programs that focus on biodiversity and soil health. The results suggest that functional groups alter resource allocation as a function of management intensity, affecting SOC, STN and related indicators.

In our study, we found that less intensive grassland management systems increase species richness mainly through having a higher proportion of legumes and forbs. This is consistent with previous studies that have shown that a grazing stocking rate of less than 1.5 livestock units per hectare promotes grassland heterogeneity and increases both floristic and faunal diversity through selective defoliation, trampling and nutrient cycling [39,40]. Overall species richness decreases significantly with increasing stocking [41,42] by reducing water availability, and high grazing intensity alters functional group composition [10]. Our results suggest that although legumes contribute to biodiversity, a higher proportion of legumes (above 17%) has a negative impact on SOC. Similar observations were made by Rodríguez et al. [35], especially when grasses and forbs were present in equal proportions. This may be explained by a reduction in community root biomass [43] and lower carbon allocation in belowground compartments when nitrogen is readily available [28]. In addition, the high metabolic activity and rapid tissue turnover of legumes may stimulate microbial decomposition through the priming effect of the rhizosphere [44]. Their nitrogen-rich litter with a low C:N ratio also decomposes rapidly [45], potentially limiting the formation of stable carbon in the soil. In addition, biological nitrogen fixation may decrease with high legume proportions due to there being less competition for soil nitrogen [33].

The response of functional groups and SOC to grazing is highly context-specific and depends on the interacting agroecological, edaphic and climatic conditions [32]. The significant increase in legumes under less intensive management is likely due to a combination of factors, including better light availability [46,47,48], lower selective grazing pressure [49,50] and lower fertilization intensity [51]. As expected, the LGI and Cut3 treatments had the highest proportion of legumes, which was most likely due to the lack of intensive fertilization, the lower selectivity of grazing, and the reduced competitive ability of grasses, which allowed legumes to thrive and add nitrogen to the soil through symbiosis with Rhizobium bacteria. Nevertheless, these two less intensive systems also had the highest C/N ratios measured at all three depths, which is statistically significant. This can be attributed to the slower decomposition of organic matter due to a lower nitrogen supply from external sources, the accumulation of lignified biomass and a lower mineralization intensity [52]. Although the presence of legumes enriches the soil with nitrogen, the effects of this process are not outweighed by the slow decomposition of plant residues with a higher carbon content, which is typical of extensive systems [22]. These results highlight the complex relationship between legume cover, cropping density and soil nutrient dynamics.

Our results showed that a lower-intensity system (LGI, Cut3) significantly increased the C:N ratio, while a more intensive management system had a significantly lower ratio. These results are in agreement with a study that found that light grazing significantly increased the soil C:N, C:P and N:P ratios, while heavy grazing decreased these ratios, which could be due to an increase in C-rich root exudates induced by light grazing [53]. In contrast, heavy grazing with frequent trampling and reduced productivity can reduce litter input and root exudation, thus leading to carbon and nitrogen losses from the soil [54,55]. The sharp increase in the C/N ratio observed under LGI and Cut3 indicates the accumulation of carbon-rich residues with limited nitrogen content. This stoichiometric imbalance can lead to microbial nitrogen immobilization as microbes sequester available nitrogen to decompose carbon-rich substrates, thereby slowing decomposition and reducing nitrogen availability to plants [56,57]. This was also confirmed by our research, in which management intensity was shown to have no significant effect on STC_stock_ but both were higher in less intensively managed systems than in more intensive managed systems. Furthermore, the published results support the notion that a higher root biomass was found on diverse, less intensively managed pastures than on conventional, intensively managed pastures [58,59].

The effects of grazing intensity on SOC have been much better studied than the effects of cutting intensity. However, overgrazing in semi-arid areas, which is associated with greatly reduced vegetation cover and soil erosion, is at the focus of many studies reporting negative effects of intensive grazing. As a global meta-analysis shows, the actual effects appear to be context-specific [32,55]. Our results suggest that management systems in which more fertilizer was applied increased the proportion of grasses compared to less intensive systems. These results are consistent with a study [21] that found a lower proportion of grasses in unfertilized treatments than in fertilized grasslands.

The intensity of grassland management affects the proportion of grasses, with the highest SOC values observed in grass-dominated systems. Higher grazing intensity contributes positively to SOC [6], which reaches peak values in grasslands with 100% grasses [35]. This result is confirmed by our data, which shows that both the proportion of grasses and SOC tend to increase in more intensive systems compared to less intensive systems, although the relationship is relatively weak (r = 0.280) and only marginally significant (*p* = 0.066). Based on the proportion of plant species, SOC_stock_ and STN_stock_ reached higher values under the dominance of grasses in the more intensive systems, with STN_stock_ reaching significant values. In more intensive systems, the presence of grasses can lead to improved stock levels [60] as they contribute organic matter via their root systems that efficiently take up soil nitrogen and decaying plant matter [47,61]. In our study, a higher proportion of legumes was associated with a lower STN_stock_ value, which is probably due to less nitrogen competition in soil with high legume dominance, which suppresses biological nitrogen fixation and thus limits the total nitrogen input [33]. In addition, increased legume biomass and root exudates stimulate microbial activity, which accelerates the mineralization of available organic nitrogen in the soil and potentially reduces total nitrogen stocks in the soil despite higher nitrogen fixation [35]. In mixtures, grasses stimulate nitrogen fixation and the transfer of fixed nitrogen from legumes, thus improving the nitrogen cycle. With a lower proportion of grasses, this positive interaction is weakened, thus resulting in less nitrogen accumulation in the soil [62,63].

Our results showed that the Shannon index was the highest on Cut4, which could be due to the fact that cutting is a less aggressive type of land use [38]. This management allows for a more balanced coexistence of species by reducing selection pressure and disturbance intensity compared to grazing, thus promoting both species richness and evenness, which are important components of the Shannon index. Grazing involves trampling and selective defoliation, which can alter the condition of functional groups and lead to a reduction in species diversity [64]. Our results are consistent with published studies [65] which report that species richness was higher in cutting plots than in grazed plots. Combined management is preferable to grazing and mowing alone, as grazing periods in autumn leave gaps in the grassland where seeds can germinate [22].

### 3.2. Grassland Management Intensity: Effects on Soil Organic and Inorganic Carbon Storage

Studies have shown that SOC is highly sensitive to changes in management systems. Conant et al. [6] reported an average SOC increase of 0.28 Mg C ha^−1^ yr^−1^ following improved grazing management, based on a global synthesis. However, they noted that responses vary depending on climate, soil, vegetation and management type [6,11]. In the Central Alps, heavy grazing increased SOC compared to light grazing and cutting [15]. In our study, CG tended to increase SOC content in the first 0–10 cm of soil, followed by LGI and HGI, compared to the other cutting systems. This trend could reflect biologically meaningful processes. The positive effects of combined grazing and mowing on soil organic carbon (SOC) can be attributed to the complementary benefits of both systems. Cutting alone is often associated with lower litter quality and slower decomposition rates compared to grazing, mainly due to reduced soil moisture and restricted nutrient cycling [14]. In addition, intensive cutting can reduce photosynthetic input, increase soil surface temperatures and stimulate soil respiration [66]. On the other hand, cutting can also promote plant community stability and support greater aboveground biomass production, which can increase overall productivity, although not necessarily SOC storage [2]. In contrast, moderate grazing increases photosynthetic efficiency through defoliation, improves root exudation and stimulates microbial activity and soil aggregation—all conditions that favor the stabilization and long-term storage of carbon in the soil [67]. We found that for HGI and Cut4, the amount of SOC and STN at the first two depths is statistically different from the amount at the third depth. This pattern can be attributed to the proportion of grasses contributing to increased carbon input from plant material [60]. In addition, the higher grass cover in our intensive systems helped to mitigate the rainfall-induced erosion of soil carbon [68]. Controlled high grazing (CHG) also resulted in higher soil compaction via livestock, which decreases soil porosity and reduces oxygen and CO_2_ exchange between the atmosphere and soil, thus limiting organic matter decomposition and soil carbon fluxes, as previous studies have shown [60]. In a CHG system, the higher percentage of manure can become an SOM potential process that builds up carbon in the soil.

Similarly, our results show that SOC_stock_ concentration at a depth of 0–30 cm tended to be the highest in CG followed by HGI and Cut4 and the lowest in the LGI and Cut3 systems. These results are supported by recent studies showing that SOC and STN were higher at a higher grazing intensity than at a lower one because frequent grazing leads to a higher deposition level of herbivore excreta [14,69]. With high grazing intensity, more forage is harvested from cows and converted into manure, which has a high potential to become organic matter in the soil, which is another potential process for soil carbon formation [3]. The SOC stock tends to increase to 1 Mg C ha^−1^ yr^−1^ through improved grazing management, fertilization, the sowing of legumes and improved grass species and irrigation [6]. Phukubye et al. [3] reported that the best management practice for grasslands is controlled rotational grazing with high grazing density, thus resulting in an average increase in soil carbon of 5.9% yr^−1^. The addition of nitrogen stabilized more aboveground residues in the soil, which could also explain some of the observed differences in SOC [19].

Globally, soils contain almost equal amounts of inorganic and organic carbon. The SIC in the upper soil profile may be more dynamic than its crystalline nature suggests [70], but in the form of calcium carbonate, it has a longer mean residence time than most SOC [71]. On average, SIC stocks were 15% higher in the top 30 cm of soil. Agricultural practices have a profound impact on the inorganic carbon cycle in soils, with soil acidification—an almost universal consequence of agriculture—having the potential to accelerate carbonate dissolution [70]. Soil management, especially irrigation and fertilization, can influence the SIC cycle and affect the ecosystem carbon cycle. Frequent N fertilization and organic acid production via roots can lower soil pH, acidify the soil and significantly reduce or dissolve SIC over time [72]. This is consistent with a study showing that composting, which increases SOC, can lead to acidification and the loss of SIC in the eight years following its initial application, especially in semi-arid regions [73]. Our results support this explanation and show that more intensive treatment is associated with lower SIC values. Although no statistically significant differences were found, the highest percentage of SIC was observed in the detached depth range of 0–30 cm in the Cut3 and LGI systems. A similar pattern was observed for SIC abundance at a 30 cm depth. Among the more intensive land uses, CG had the highest proportion of SIC. Management practices aimed at increasing SOC sequestration could potentially reduce SIC stocks. In some cases, this reduction in SIC may lead to the release of CO_2_ into the atmosphere, depending on the specific dissolution processes affecting this inorganic carbon [71]. A limited number of studies have investigated the effects of grazing and cutting on soil inorganic carbon (SIC). The influence of grazing intensity on soil pH and nutrient dynamics is highly dependent on soil type, and identical management practices on different soils can lead to different results [74]. Grazing can alter the soil microbial community and nutrient availability by recycling animal manure and urine, thereby influencing soil properties [75]. Reeder et al. [76] suggested that grazing can affect SIC by increasing the production of organic acids in root exudates, which accelerate the weathering of calcium-containing minerals and release Ca^2+^ into the soil profile. In addition, CO_2_ from root respiration reacts with water to form bicarbonate (HCO_3_^−^), which then combines with the Ca^2+^ released from weathered minerals. In contrast, cutting can also affect soil pH, mainly through its effects on biomass decomposition and the duration and intensity of cutting [77]. Therefore, the higher SIC observed in less intensive systems could result from the complementary effects of moderate grazing and cutting systems that promote carbonate precipitation and less disturbance in cutting systems, keeping the pH and mineral balance stable.

### 3.3. Nitrogen and Phosphorus Responses to Different Grassland Management Intensities

Numerous field studies have investigated the effects of grazing intensity on STN with mixed results [78]. However, on average, moderate grazing intensity was found to result in higher rates of STN compared to ungrazed or heavily grazed systems. In our study, we can speculate whether the increase in the mineralization of SOM measured in CG, Cut4 and HGI was the result of an increase in STN and SOC or an increase in the proportion of grasses. This hypothesis was further supported by the correlation between grasses and SOC (*p* = 0.066; r = 0.280). These results are consistent with published studies [79,80] reporting that grazing intensity increases STN. The effects of the combined management system are similar to those of grazing alone, thus suggesting that mowing already grazed grassland has no additional negative effects on functions. The combined approach of grazing and cutting can optimize nitrogen availability by balancing the decomposition of biomass and the recycling of nutrients through animal waste [38].

The P concentrations in our study were low, especially the highest concentrations in both grazing systems at the first depth. These results are in agreement with a published study [53] that reported that grazing increases the soil phosphorus pool by 3.6%. Large amounts of phosphorus are returned to the soil via manure and urine [13]. Manure is a source of labile phosphorus that can increase microbial biomass, and intensive grazing can influence phosphorus losses [81]. Delgado-Baquerizo et al. [82] reported that phosphorus is mainly derived from mineral weathering. They therefore hypothesized that grazing increases phosphorus availability by stimulating the rate of rock weathering, possibly by reducing plant cover and soil exposure. Precipitation also influences the soil phosphorus pool, with grazing increasing soil phosphorus content by 2.3% when the MAP (mean annual precipitation) is 400–800 mm and decreasing it by 4.2% when the MAP is <400 mm [70]. The latter is also confirmed by our results. The highest soil phosphorus content was found in the first 0–10 cm depth in both grazing systems.

In our study, management intensity affected the composition of functional groups and the chemical properties of soil. Less intensive systems supported a higher proportion of legumes, while species diversity peaked under the Cut4 regime. Combined management strategies increased the SOC and STN stocks, especially in the CG, Cut4 and HGI systems. Adapting management intensity to local climatic and environmental conditions is crucial to improve carbon sequestration and maintain ecosystem stability. To better understand the long-term effects of management on soil properties and organic matter turnover, future studies should include information on deeper soil layers and microbial or enzymatic activities.

## 4. Materials and Methods

### 4.1. Experimental Site and Study Design

The trial site was located in eastern Slovenia (from 46°33′07.5″ N, 14°53′50.3″ E to 46°28′26.7″ N, 15°59′58.9″ E) in an area where 72% of all livestock farms are concentrated. Prior to sampling in 2020, individual interviews were conducted with the owners of all farms to obtain detailed information on the history of land use and farming systems. The selected dairy farms described in our previous study [37] are characterized by permanent grassland and have been continuously farmed for more than 30 years. This long-term continuity of land use is crucial as it promotes the development of a stable soil structure, improves the accumulation of soil organic matter and supports a diverse and resilient soil microbial community, all of which are fundamental to maintaining soil health and biodiversity in grassland ecosystems. They were selected based on specific pedoclimatic characteristics, including the presence of eutric cambisols (textural classes: silty loam, silty loam and silty clay).

For the experimental design, 45 farms were selected. These farms were located in lowland areas with a temperate climate at an altitude between 260 and 390 m above sea level. They were specifically selected for their influence on the uniformity of botanical composition, which allowed for a more appropriate and targeted analysis. During the 2020 growing season (April to October), the average annual air temperature was 10.3 °C, and the average annual precipitation was 957 mm. All measurements were taken in autumn 2020, after the end of the growing season. Five different grassland management systems were included in this study: (1) the Cut3 system (three cuts per year); (2) the Cut4 system (four cuts per year); (3) a system with low grazing intensity (LGI), in which the livestock density is below 1.6 livestock units per hectare; (4) a system with high grazing intensity (HGI), in which the livestock density is above 1.6 livestock units per hectare; and (5) combined cutting and grazing (CG), in which management involves two or three cuts in summer followed by grazing in autumn. These systems were selected to reflect a realistic gradient of management intensity and the combinations of cutting and grazing that are common in the region studied.

### 4.2. Field Sampling and Measurements

Soil samples were collected from 45 farms representing the typical range of grassland use in the region across the five management systems studied: Cut3 (14 farms), Cut4 (11 farms), LGI (6 farms), HGI (7 farms) and CG (7 farms). Fertilization practices varied between the management systems. In the cut systems, 10 m^3^ ha^−1^ of slurry was applied after each cut, while in the CG system, 15 m^3^ ha^−1^ of slurry was applied after each cut, except after the last cut. No additional fertilizer was applied to the pastures where the dairy cows grazed daily from mid-April to mid-October. In all systems, phosphorus mineral fertilizer was only applied in spring, during the first fertilization. In the cutting systems, 40 kg P_2_O_5_ ha^−1^ per year was applied, while in the grazing systems, 70 kg P_2_O_5_ ha^−1^ per year was applied.

The functional group composition of the grassland was determined via a direct visual estimation, in which three observers simultaneously assessed the proportion of individual plant species and functional groups (grasses, legumes and forbs) within each sample plot [83]. The estimates were treated as independent replicates, and consistency was ensured using a standardized protocol and real-time consensus in the field. The values of the most commonly used parameters for assessing grassland species diversity were calculated. The formulae used to calculate the diversity measures correspond to those of Morris et al. [84]:

Species richness (S) = number of species,(1)

(2) Shannon’s diversity index (H)=−∑pilnpi

*pi*—the percentage of species cover of particular species.

### 4.3. Laboratory Analyses

Soil samples were also taken at all sites, including three replicates for physical properties and ten replicates for chemical properties. The soil samples for a soil analysis of SOC, SIC, STC, STN, SOM, pH, P_2_O and K_2_O were taken from a depth of 30 cm and divided into three layers: 0–10 cm, 10–20 cm and 20–30 cm. The samples were taken with a soil auger with a diameter of 1 cm, and ten subsamples were randomly distributed. The BD of the soil was measured at each depth in three replicates (first, second and third depths) using the core method. POR was calculated from the known BD and the density of soil particles (2.65 g cm^−3^) [85]. The determination of the VWC was also based on this method with the additional determination and calculation of the gravimetric water content (W). The pH value was determined according to the international standard ISO 10390, which specifies an instrumental method for the routine determination of the pH value with a glass electrode in a 1:5 (volume fraction) soil suspension in water. The amounts of ammonium lactate-soluble P_2_O_5_ and K_2_O (mg per 100 g of air-dried soil) were determined according to Egner et al. [86]. The soil samples for the determination of SOC, SIC, STN and SOM were analyzed at the Alice Holt Research Station, UK. Prior to analysis, plant material and stones were removed, and the samples were air-dried, ground, sieved (2 mm), ball-milled and homogenized. STC and STN were measured according to ISO standards 10694 and 13878 using a Carlo Erba CN Flash EA1112 analyzer (Wigan, UK). For each test, 30 mg of soil was combusted at 900 °C in oxygen with an oxidation catalyst. Carbon and nitrogen were then separated via gas chromatography and detected thermally. SIC was determined by heating subsamples at 500 °C for 2 h to remove organic carbon. SOC was calculated as the difference between STC and SIC. Further details on all calculations of the targeted soil properties for each depth layer from 0 to 30 cm are described in [37]. We also determined the sum of total carbon (STC_stock_), organic carbon (SOC_stock_) and total nitrogen (STN_stock_) in the soil at the individual depths of 0–30 cm. SOM was calculated for each depth (i) using the following formula:

SOM_i_ (%) = Corg_i_ × 1.724 (3)

### 4.4. Statistical Analyses

Statistical analyses were performed using the Statistical Package for the Social Sciences (IBM SPSS 22.0, Chicago, IL, USA). Linear mixed models were used to analyze the effects of the investigated factors (grassland management and soil depth) on different physical and chemical properties. In the models that used physical (VWC, POR, BD) and chemical soil properties (SOC, STN, STC, C/N, SOM, pH, P_2_O_5_, K_2_O) as dependent variables, grassland management, soil depth and their interaction were included as fixed factors and elevation and precipitation as random effects. The percentage composition of the functional groups (grasses, legumes and forbs) and the two indices (species richness and Shannon diversity) were also determined using the same model. Generalized gamma regression was used to analyze SIC. Estimated means with standard errors of the mean for the factors and interactions analyzed were calculated for the generated models. Post hoc comparisons were adjusted using the Bonferroni correction for multiple tests. A visual inspection of the residual plots for the models revealed no obvious deviations from homoscedasticity or normality. The Pearson correlation coefficient was used to analyze the relationship between functional groups (grasses, legumes and forbs) and some chemical properties (SOC, STN, SOM, STC, P_2_O_5_, K_2_O).

## 5. Conclusions

This study provides evidence that the intensity of grassland management significantly influences the composition of plant functional groups and alters important chemical properties of the soil. Among the systems studied, combined cutting and grazing proved to be the most effective strategy for increasing soil organic carbon and total nitrogen, thus indicating its potential for the sustainable management of semi-natural grasslands.

The results show the importance of adapting management intensity to local environmental conditions to promote nutrient cycling, improve forage productivity and strengthen ecosystem resilience. The remarkable contribution of soil inorganic carbon at a depth of more than 20 cm also emphasizes the need for integrated carbon accounting that includes both organic and inorganic carbon pools.

These results have important implications for evidence-based conservation and restoration strategies. In particular, they can influence agri-environmental policies by emphasizing management approaches that promote soil carbon sequestration and biodiversity. For example, Common Agricultural Policy (CAP) reforms or regional subsidy schemes could favor combined cutting and grazing systems in areas where soil health and ecosystem services are critical. Focusing financial support on these goals would strengthen efforts to mitigate climate change and promote long-term sustainability.

Future research should focus on long-term monitoring and clarifying the functional role of plant species in shaping soil carbon dynamics under different management regimes, including deeper soil layers and biological indicators such as microbial or enzymatic activity.

## Figures and Tables

**Table 1:** Proportions of functional groups and diversity indices under different grassland management systems (presented as means ± standard errors).

Management System	Legumes (%)	Grasses (%)	Forbs (%)	S	H
**CG**	10.42 ± 2.26 ab	63.03 ± 6.10 a	25.59 ± 4.42	24.42 ± 2.82	2.99 ± 0.16 b
**Cut3**	13.51 ± 1.92 a	56.00 ± 5.68 b	30.00 ± 3.66	27.34 ± 2.14	2.78 ± 0.13 bc
**Cut4**	6.63 ± 2.13 bc	62.86 ± 5.91 a	29.13 ± 4.03	25.94 ± 2.50	3.33 ± 0.14 a
**LGI**	15.03 ± 2.46 a	45.51 ± 6.28 c	37.26 ± 4.78	30.31 ± 2.86	2.84 ± 0.15 bc
**HGI**	5.38 ± 2.28 c	60.98 ± 6.18 a	32.40 ± 4.51	22.34 ± 2.84	2.50 ± 0.16 c
*p*-value	<0.001	0.009	0.289	0.286	<0.001

(a, b, c) Different letters indicate significantly different means based on a Bonferroni test with *p* < 0.05 for each management type. Management systems: CG—combined cutting and grazing; Cut3—three cuts per year; Cut4—four cuts per year; LGI—low grazing intensity; HGI—high grazing intensity. S—species richness (number of plant species); H—Shannon diversity index.

**Table 2:** The effects of the management system and soil depth on the analyzed physico-chemical properties of the soil (presented as means ± standard errors).

	Management System						Soil Depths			
Soil Properties	CG	Cut3	Cut4	LGI	HGI	*p*-Value	0–10	10–20	20–30	*p*-Value
**Physical**										
**VWC**	37.62 ± 3.73	40.68 ± 3.54	37.96 ± 3.63	39.25 ± 3.93	41.94 ± 3.74	0.285	43.54 ± 3.52	38.72 ± 3.52	36.21 ± 3.52	≤0.001
**POR**	46.77 ± 3.44	48.14 ± 3.18	43.85 ± 3.31	46.18 ± 3.69	48.49 ± 3.46	0.278	55.51 ± 3.17	44.29 ± 3.17	40.26 ± 3.14	≤0.001
**BD**	1.26 ± 0.05	1.22 ± 0.04	1.26 ± 0.05	1.25 ± 0.06	1.24 ± 0.05	0.834	1.05 ± 0.05	1.31 ± 0.04	1.39 ± 0.05	≤0.001
**Chemical**										
**SOC**	27.56 ± 2.47	22.86 ± 2.24	27.04 ± 2.36	23.81 ± 2.62	27.37 ± 2.48	0.037	33.63 ± 2.24	24.58 ± 2.23	18.98 ± 2.24	≤0.001
**SIC**	2.11 ± 0.69	17.26 ± 4.42	1.58 ± 0.58	14.76 ± 6.71	-	≤0.001	4.16 ± 1.38	5.48 ± 1.66	6.92 ± 2.06	0.523
**STC**	25.15 ± 2.99	28.74 ± 2.68	26.24 ± 2.81	29.70 ± 3.06	26.07 ± 2.98	0.427	34.52 ± 2.67	26.08 ± 2.66	20.94 ± 2.67	≤0.001
**STN**	2.80 ± 0.21	2.28 ± 0.17	2.94 ± 0.19	2.23 ± 0.23	2.87 ± 0.21	0.002	3.45 ± 0.17	2.55 ± 0.16	1.87 ± 0.17	≤0.001
**P**	1.02 ± 0.35	1.17 ± 0.27	1.01 ± 0.29	1.53 ± 0.38	1.79 ± 0.35	0.282	1.86 ± 0.27	0.99 ± 0.26	1.05 ± 0.26	0.012
**K**	8.57 ± 5.15	11.81 ± 4.79	14.10 ± 4.98	12.98 ± 5.47	12.27 ± 5.17	0.591	16.66 ± 4.79	10.41 ± 4.79	8.78 ± 4.78	0.004
**SOM**	4.53 ± 0.46	3.92 ± 0.42	4.23 ± 0.45	3.90 ± 0.49	4.32 ± 4.46	0.407	6.15 ± 0.42	3.65 ± 0.43	2.74 ± 0.42	≤0.001
**pH**	6.07 ± 0.51	6.63 ± 0.49	6.06 ± 0.50	6.59 ± 0.52	6.06 ± 0.51	0.945	6.26 ± 0.49	6.28 ± 0.49	6.31 ± 0.49	0.013
**C/N**	7.46 ± 3.34	13.78 ± 3.19	7.34 ± 3.27	20.13 ± 3.44	6.89 ± 3.35	≤0.001	9.54 ± 3.18	10.42 ± 3.17	13.41 ± 3.18	0.010

SOC—soil organic carbon; SIC—soil inorganic carbon; STC—soil total carbon; STN—soil total nitrogen; P—phosphorus pentoxide (P_2_O_5_); K—potassium oxide (K_2_O); SOM—soil organic matter; VWC—volumetric soil water content; BD—bulk density; POR—porosity; C/N—carbon/nitrogen ratio. The interaction between the management system and soil depth was insignificant for all physical and chemical properties of the soil except for C/N.

**Table 3:** The physical properties of the soil under five different grassland management systems at three depths (0–10, 10–20 and 20–30 cm) (presented as means ± standard errors).

	Soil Depth (cm)			
Management System	0–10	10–20	20–30	*p*-Value
**VWC (%)**				
CG	38.06 ± 6.60	37.31 ± 3.80	37.48 ± 3.35	0.980
Cut3	46.14 ± 5.09 a	38.87 ± 3.28 b	37.04 ± 2.93 b	0.005
Cut4	42.95 ± 5.29 a	36.77 ± 3.38 a	34.15 ± 3.06 b	0.017
LGI	43.01 ± 6.09	40.48 ± 4.28	34.06 ± 3.73	0.106
HGI	47.52 ± 5.63 a	40.17 ± 3.86 ab	38.11 ± 3.39 b	0.044
*p*-value	0.131	0.827	0.746	
**POR (%)**				
CG	54.97 ± 4.31 a	45.81 ± 3.93 b	39.54 ± 3.42	0.002
Cut3	60.43 ± 3.53 a	42.46 ± 3.37 b	41.53 ± 2.63	<0.001
Cut4	52.12 ± 3.74 a	41.45 ± 3.56 b	37.98 ± 2.86	<0.001
LGI	54.40 ± 4.91 a	45.55 ± 4.39 ab	38.59 ± 3.91	0.004
HGI	55.62 ± 4.38 a	46.19 ± 3.98 b	43.67 ± 3.47	0.015
*p*-value	0.163	0.636	0.625	0.002
**BD (g cm^−3^)**				
CG	1.08 ± 0.09 b	1.31 ± 0.06 a	1.38 ± 0.05 a	<0.001
Cut3	0.94 ± 0.07 b	1.35 ± 0.05 a	1.38 ± 0.04 a	<0.001
Cut4	1.08 ± 0.07 b	1.31 ± 0.05 a	1.40 ± 0.04 a	<0.001
LGI	1.07 ± 0.98 b	1.27 ± 0.07 a	1.43 ± 0.06 a	<0.001
HGI	1.08 ± 0.08 b	1.30 ± 0.06 a	1.36 ± 0.05 a	0.001
*p*-value	0.094	0.816	0.937	

(a, b): Different letters indicate significantly different means based on a Bonferroni test with *p* < 0.05, for each management system within the different depths. VWC—volumetric soil water content; BD—bulk density; POR—porosity. Management systems: CG—combined cutting and grazing; Cut3—three cuts per year; Cut4—four cuts per year; LGI—low grazing intensity; HGI—high grazing intensity.

## Data Availability

The data may be made available from the authors upon reasonable request.
